# The Integration of Radiomics and Artificial Intelligence in Modern Medicine

**DOI:** 10.3390/life14101248

**Published:** 2024-10-01

**Authors:** Antonino Maniaci, Salvatore Lavalle, Caterina Gagliano, Mario Lentini, Edoardo Masiello, Federica Parisi, Giannicola Iannella, Nicole Dalia Cilia, Valerio Salerno, Giacomo Cusumano, Luigi La Via

**Affiliations:** 1 Faculty of Medicine and Surgery, University of Enna “Kore”, 94100 Enna, Italy; antonino.maniaci@unikore.it (A.M.); salvatore.lavalle@unikore.it (S.L.); caterina.gagliano@unikore.it (C.G.); 2 ASP Ragusa, Hospital Giovanni Paolo II, 97100 Ragusa, Italy; marlentini@tiscali.it; 3 Radiology Unit, Department Clinical and Experimental, Experimental Imaging Center, Vita-Salute San Raffaele University, 20132 Milan, Italy; 4 Department of Medical and Surgical Sciences and Advanced Technologies “GF Ingrassia”, ENT Section, University of Catania, Via S. Sofia, 78, 95125 Catania, Italy; federicamariaparisi@gmail.com; 5 Department of ‘Organi di Senso’, University “Sapienza”, Viale dell’Università, 33, 00185 Rome, Italy; giannicola.iannella@uniroma1.it; 6 Department of Computer Engineering, University of Enna “Kore”, 94100 Enna, Italy; nicoledalia.cilia@unikore.it; 7 Institute for Computing and Information Sciences, Radboud University Nijmegen, 6544 Nijmegen, The Netherlands; 8 Department of Engineering and Architecture, Kore University of Enna, 94100 Enna, Italy; valerio.salerno@unikore.it; 9 University Hospital Policlinico “G. Rodolico—San Marco”, 95123 Catania, Italy; giacomo.cusumano@unict.it; 10 Department of General Surgery and Medical-Surgical Specialties, University of Catania, 95123 Catania, Italy

**Keywords:** radiomics, artificial intelligence, medical imaging, predictive modeling, personalized medicine

## Abstract

With profound effects on patient care, the role of artificial intelligence (AI) in radiomics has become a disruptive force in contemporary medicine. Radiomics, the quantitative feature extraction and analysis from medical images, offers useful imaging biomarkers that can reveal important information about the nature of diseases, how well patients respond to treatment and patient outcomes. The use of AI techniques in radiomics, such as machine learning and deep learning, has made it possible to create sophisticated computer-aided diagnostic systems, predictive models, and decision support tools. The many uses of AI in radiomics are examined in this review, encompassing its involvement of quantitative feature extraction from medical images, the machine learning, deep learning and computer-aided diagnostic (CAD) systems approaches in radiomics, and the effect of radiomics and AI on improving workflow automation and efficiency, optimize clinical trials and patient stratification. This review also covers the predictive modeling improvement by machine learning in radiomics, the multimodal integration and enhanced deep learning architectures, and the regulatory and clinical adoption considerations for radiomics-based CAD. Particular emphasis is given to the enormous potential for enhancing diagnosis precision, treatment personalization, and overall patient outcomes.

## 1. Introduction

**Figure 1:**
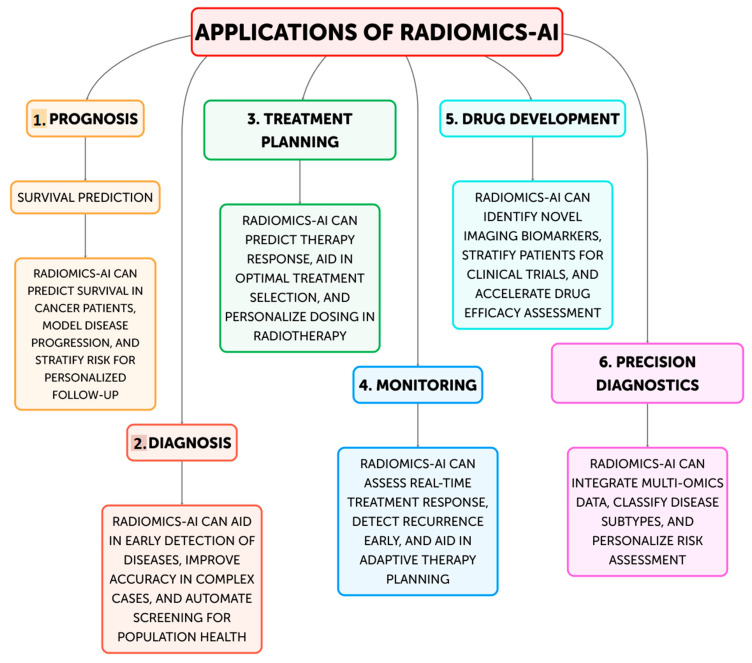
Current and future applications of Radiomics-AI.

In order to improve clinical decision-making and customized medicine, the discipline of radiomics—the extraction and analysis of quantitative information from medical images—has drawn a lot of interest recently [1,2,3]. This data-driven approach makes use of cutting-edge computational techniques to find potential imaging biomarkers and hidden patterns in medical scans, which can provide important information about the nature of the disease, how well a treatment is working, and how patients will fare [4]. The area of radiology has undergone a revolution with the incorporation of artificial intelligence (AI) techniques, including machine learning and deep learning [5,6]. AI-driven systems have proven to be able to identify clinically important imaging biomarkers more easily, automate workflows, and increase diagnostic accuracy (Figure 1) [7,8]. The combination of AI with radiomics has the potential to change medical imaging from a qualitative to a quantitative field, allowing for more individualized and data-driven patient care strategies [9,10].

### Aim of the Review

The goal of this paper is to give a thorough overview of how AI and radiomics are now being integrated in contemporary medicine. In particular, this review examines the many uses of AI in radiomics, including its involvement of quantitative feature extraction from medical images, the machine learning, deep learning and computer-aided diagnostic (CAD) systems approaches in radiomics, and the effect of radiomics and AI on improving workflow automation and efficiency, optimize clinical trials and patient stratification. This paper also covers the predictive modeling improvement by machine learning in radiomics, the multimodal integration and enhanced deep learning architectures, and the regulatory and clinical adoption considerations for radiomics-based CAD.

## 2. Materials and Methods

The authors performed a thorough electronic literature search on Medline, Embase, Cochrane and PubMed up to 1 May 2024. The keywords “radiomics”, “artificial intelligence”, “machine learning”, “deep learning” were searched and combined using Boolean operators, together with the words “healthcare” and “medical imaging”. This extensive search strategy made sure that a wide range of pertinent publications, from foundational works to the most recent developments in the field, were included in the review. Many articles that might have been of interest were found during the first search; these were then put through a stringent screening procedure. In order to aid in the selection of studies, the authors developed a set of inclusion criteria. They concentrated on articles that offered thorough summaries or in-depth discussions of radiomics and its integration with AI, as well as studies that illustrated the applications of these technologies with a focus on cardiology, neurology, and oncology. The evaluation examined how AI and radiomics are affecting important fields like clinical trials, workflow automation, predictive modeling, and personalized medicine. Following the preliminary screening, a thorough review of the chosen articles was conducted, and pertinent data was extracted and arranged.

## 3. Results

Our literature search on Medline, Embase, Cochrane and PubMed identified 10 key studies (Table 1) that highlight the integration of radiomics and artificial intelligence in medical imaging across various cancer types and imaging modalities.

Moreover, several other studies were included on the current and future possible applications of radiomics-AI in modern medicine. Figure 1 provides a summary of the six main applications of radiomics-AI, that will be further discussed in the following paragraphs.

## 4. Radiomics and Its Involvement of Quantitative Feature Extraction from Medical Images

### 4.1. Introduction

**Figure 2:**
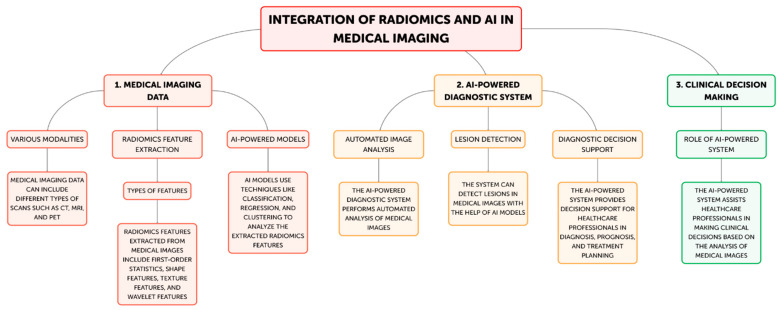
Steps of radiomics-AI processes in medical imaging. Step 1A: Various modalities; Step 1B: Radiomics feature extraction; Step 2A: Automated image analysis; Step 2B: Lesion Detection; Step 2C: Diagnostic decision support; Step 3: Clinical decision making.

Radiomics has become a potent technique that uses cutting-edge computational techniques to extract and analyze a significant amount of quantitative information from medical images [2]. These traits, which are also known as imaging biomarkers, might offer important insights into the underlying traits of various illnesses, including tumor phenotype, genetic profiles, and responsiveness to treatment [3]. Radiomics has been used in oncology, neurology, and cardiology, among other medical specialties, with the goal of improving disease diagnosis, prognosis, and treatment personalization [9,10]. Radiomics normally entails a number of important steps (Figure 2).

### 4.2. Discussion

The first step in the radiomics process is usually obtaining medical images from computed tomography (CT), magnetic resonance imaging (MRI), and positron emission tomography (PET). After that, these images go through preprocessing procedures designed to improve uniformity and caliber among datasets [1,2]. Subsequently, an extensive array of quantitative features, encompassing intensity-based features, texture features, form features, and spatial connection features, are retrieved from the images. These properties capture the variability, appearance, and functional characteristics of the imaged tissues [4]. Advanced computational techniques, including machine learning algorithms, are employed to assess the collected data and identify patterns and connections that could potentially indicate disease characteristics, treatment response, or patient outcomes [10]. Building upon these foundational steps, advanced radiomics incorporates automated image analysis techniques to further enhance the extraction and interpretation of medical imaging data. Sophisticated algorithms can automatically segment regions of interest, reducing human variability and increasing efficiency in image processing [2]. This automated approach is particularly valuable in lesion detection, where AI-powered systems can rapidly scan large volumes of imaging data to identify potential abnormalities with high sensitivity and specificity [6]. In the realm of diagnostic decision support, radiomics features can be integrated with clinical data and other biomarkers to create comprehensive predictive models. These models can assist radiologists and clinicians in differentiating between benign and malignant lesions, grading tumors, or predicting the likelihood of specific diagnoses [1]. Such tools can potentially reduce inter-observer variability and improve diagnostic accuracy. Furthermore, radiomics is increasingly being applied to clinical decision making. By correlating radiomic signatures with treatment outcomes, these advanced analytical methods can guide personalized treatment strategies. For instance, radiomics-based models can predict treatment response in oncology, helping clinicians choose between different therapeutic options or adjust treatment plans based on early response indicators [3]. This approach aligns with the goals of precision medicine, where treatment decisions are tailored to individual patient characteristics. For example, research has shown how radiomics-derived features can be used to track the evolution of different cancer types, detect certain genetic changes, and forecast the chance of tumor recurrence [8]. By detecting minor changes in the structure and function of the brain, radiomics has been used in the field of neurology to aid in the identification of neurodegenerative diseases, including Alzheimer’s disease [6]. The possibility for non-invasive, quantitative insights from imaging biomarkers produced from radiomics to supplement conventional diagnostic techniques accounts for their clinical significance [1]. These biomarkers can be useful instruments for personalized medicine, empowering medical professionals to choose treatments, manage patients, and manage diseases with more knowledge [3,4]. Through the application of radiomics, healthcare professionals can transcend the qualitative evaluation of medical imaging and adopt a more objective, data-driven approach to m. Additionally, the capabilities of this approach have been further strengthened by the integration of radiomics with AI approaches, such as machine learning and deep learning [5]. AI-powered solutions have proven to be able to identify clinically relevant imaging biomarkers more easily, increase diagnostic accuracy, and automate workflow operations [7,8].

### 4.3. Conclusions

The combination of AI with radiomics has the potential to change medical imaging from a qualitative to a quantitative field, allowing for more individualized and data-driven patient care strategies. Radiomics has several uses and is still developing for prognostic and diagnostic purposes. It is anticipated that as radiomics develops, it will contribute more significantly to the growth of precision and customized medicine, which will ultimately improve patient outcomes and improve clinical decision-making.

## 5. Machine Learning, Deep Learning and Computer-Aided Diagnostic (CAD) Systems Approaches in Radiomics

### 5.1. Introduction

The discipline of radiomics has undergone a revolution with the incorporation of AI techniques, including machine learning and deep learning [5]. AI-powered solutions have proven to be able to identify clinically relevant imaging biomarkers more easily, increase diagnostic accuracy, and automate workflow operations [8].

### 5.2. Discussion

In radiomics analysis, machine learning techniques have been widely used to create predictive models and decision assistance tools [5,6]. Large datasets of radiomics features and related clinical outcomes can be used to train these algorithms, enabling them to discover patterns and associations that can help with prognosis, treatment selection, and illness diagnosis [16,17]. For instance, based on radiomic characteristics taken from pre-treatment images, studies have employed machine learning algorithms to predict therapy response in cancer patients [11,18]. The discipline of radiomics has also seen notable advancements in deep learning, a branch of machine learning [6]. Convolutional neural networks (CNNs) are examples of deep learning models that may learn pertinent features directly from unprocessed image data, obviating the requirement for human feature engineering [19,20]. In many situations, these models have outperformed conventional machine learning techniques in tasks including tumor segmentation, lesion identification, and image classification [21,22]. Advanced CAD systems have been developed as a result of radiomics-AI [8]. These systems use AI to detect anomalies, automate image processing, and give doctors decision-support resources. Numerous medical applications, such as the diagnosis of lung cancer, breast cancer, and different neurological problems, have been studied with regard to CAD systems [23,24].

### 5.3. Conclusions

Medical imaging could become a more quantitative field with the combination of radiomics and AI, opening the door to more individualized and data-driven patient care strategies [5]. Clinicians can improve patient outcomes, increase diagnostic accuracy, and make better decisions by utilizing the capabilities of these tools.

## 6. The Effect of Radiomics and AI on Improving Workflow Automation and Efficiency, Optimize Clinical Trials and Patient Stratification

### 6.1. Introduction

Automating picture processing is one of the main advantages of AI-powered radiomics. With a sensitivity of 94% and specificity of 96%, Hosny et al. (2018) showed the potential of deep learning models in automating the detection of lung nodules on CT images [13]. This degree of precision increases the consistency and dependability of diagnoses while also lightening the workload for radiologists.

### 6.2. Discussion

An AI system that can triage mammograms for breast cancer screening was demonstrated by Rodríguez-Ruiz et al. (2019), potentially reducing radiologist workload by 20% without sacrificing cancer detection rates [25]. This triage method serves as an example of how AI can rank cases according to importance, freeing radiologists to concentrate their skills on more difficult or urgent cases. The process of creating reports is also automated. Natural language processing techniques have been reported by Langlotz et al. (2019) to pre-populate radiological reports, potentially saving up to 50% on reporting time [26]. This guarantees uniformity in report terminology and structure while also expediting the reporting process. An AI system that could automatically extract and quantify radiomics features from lung CT scans was demonstrated by Trivizakis et al. (2020), cutting the feature extraction process from hours to minutes [27]. There are important ramifications for clinical practice and medical research from this acceleration of research operations. AI and radiomics have had a significant impact on clinical trials, especially in the areas of outcome prediction and patient stratification. A radiomics signature with an area under the curve (AUC) of 0.86 was created by Sun et al. (2020) to predict the immunotherapy response in patients with non-small cell lung cancer [28]. This degree of predictive precision may greatly enhance the process of choosing patients for immunotherapy trials, which could result in more successful and economical clinical research. In neuro-oncology, Kickingereder et al. (2019) predicted MGMT promoter methylation status in glioblastoma patients with 88% accuracy by utilizing a machine learning model that integrated radiomics data from multiparametric MRI [29]. In brain tumor trials, this non-invasive way of evaluating molecular markers may improve patient classification and lessen the requirement for invasive biopsies. Clinical trial design will be significantly impacted by AI-driven radiomics’ capacity to find imaging biomarkers indicative of illness progression or treatment response. It makes it possible to choose patients more precisely, which could lead to smaller trial sample sizes and quicker drug development. AI and radiomics are showing value in patient stratification for customized treatment methods, even outside of clinical trials. Aerts et al. (2019) greatly outperformed traditional TNM staging (AUC 0.75) with their deep learning model for lung cancer prognostication utilizing CT images, obtaining an AUC of 0.92 for two-year survival prediction [13]. Peritumoral tissue patterns, among other hitherto unidentified imaging indicators, were found to be strongly predictive of survival by this model. Zhu et al. (2021) used a support vector machine classifier in conjunction with radiomics characteristics to predict the response to neoadjuvant chemotherapy in breast cancer [30]. Their model, which included 267 radiomics variables taken from MRI scans prior to treatment, was able to predict pathological full response with an accuracy of 89%, as opposed to 71% for traditional clinical assessment. Patient outcomes could be enhanced and healthcare resources could be used more effectively if these developments in patient classification could more precisely inform treatment decisions. Notwithstanding these encouraging advancements, obstacles still need to be overcome before AI and radiomics may be widely used in clinical settings. Up to 30% of frequently used radiomics characteristics have poor reproducibility across various CT scanners, as noted by Traverso et al. (2020), underscoring the necessity of standardizing feature extraction and imaging techniques [31]. Both amount and quality of data continue to be major obstacles. Robust AI models that can generalize across various patient groups and imaging equipment require training on large, diverse datasets [32]. Although there has been some progress, more effort has to be done in the field of creating uniform, multi-institutional databases. Another significant issue is the interpretability of AI models. It gets harder to understand the logic underlying models’ predictions as they get more complicated. Research is being done to provide insights into model decisions using techniques such as SHAP (SHapley Additive exPlanations) values, as Selvaraju et al. (2020) showed in their work on explainable AI for medical imaging [33].

### 6.3. Conclusions

Healthcare AI regulatory frameworks are continually developing. The Food and Drug Administration (FDA) has started creating rules for assessing medical devices that use AI and machine learning, but regulatory agencies have difficulties due to the speed at which technology is advancing. In the future, there will probably be a faster integration of AI and radiomics into clinical operations. More advanced AI models that can evaluate several imaging modalities at once are likely to come along, offering a more thorough evaluation of illness conditions. AI models that combine radiomics with other -omics data (genomics, proteomics, etc.) have the potential to improve patient classification and treatment planning even further.

## 7. Predictive Modeling Improvement by Machine Learning in Radiomics

### 7.1. Introduction

Predictive modeling in medical imaging has recently improved thanks to the combination of machine learning (ML) and radiomics. This section delves into state-of-the-art machine learning algorithms and their particular applications in radiomics, showcasing new research that shows notable progress in prognosis, treatment response prediction, and disease identification.

### 7.2. Discusson

CNNs have performed remarkably well in radiomics based on images. Using low-dose chest CT scans, Ardila et al. (2019) created a CNN model for lung cancer screening. On the National Lung Screening Trial dataset, their algorithm outperformed radiologists with an AUC of 0.94. In addition to identifying malignant lung nodules, CNN also offered geographical data and an overall risk assessment for lung cancer [34]. In radiomics, random forests and gradient boosting machines have shown to be successful. A random forest classifier was used by Kickingereder et al. (2019) to examine multiparametric MRI characteristics in patients with glioblastoma. The MGMT promoter methylation status is a critical indicator for treatment response, and their model predicted it with 88% accuracy [29]. This non-invasive method may lessen the requirement for invasive biopsies. Yao et al. (2020) created a deep learning model to predict overall survival in patients with non-small cell lung cancer by combining clinical data and CT radiomics features. Their methodology substantially outperformed conventional TNM staging (0.614) with a concordance index of 0.739 [35]. The potential for increased prognosis accuracy by combining radiomics with other data types is demonstrated by this study. Wu et al. (2019) used radiomic characteristics taken from breast MRI scans and applied k-means clustering on them. They discovered three unique imaging characteristics linked to several molecular subtypes of breast cancer. This unsupervised method identified novel biomarkers that might be used to tailor treatment plans [36]. High dimensionality radiomic characteristics have been addressed with Principal Component Analysis (PCA) and t-SNE. Parmar et al. (2018) conducted a comparison of several feature selection techniques in CT-based radiomics for the prognosis of head and neck cancer. They discovered that the best prognostic performance (AUC = 0.80) was obtained when PCA and random forest feature selection were combined [37]. A study by Hosny et al. (2020) shown that transfer learning works in radiomics. They trained a CNN on natural photos beforehand and optimized it for the categorization of lung nodules. With an AUC of 0.90 on a separate test set, our method demonstrated how transfer learning may be used to overcome the limitations of small medical imaging datasets [38]. In order to mitigate data privacy issues and facilitate inter-institutional cooperation, Sheller et al. (2020) developed a federated learning strategy for brain tumor classification. Their model retained patient data locally at each institution, however it performed on par with centralized learning [39]. The deep learning model developed by Gao et al. (2021) for the prediction of the Gleason score from MRI in prostate cancer included attention processes. With an accuracy of 85%, the attention-based model outperformed conventional CNN architectures and offered comprehensible visual explanations for its predictions [40]. Making sure ML models are interpretable is essential for clinical adoption as they get more complicated. In order to evaluate a random forest model that predicts lung cancer recurrence using CT radiomics data, Lundberg et al. (2020) employed SHAP values. This method increased confidence in the model’s decision-making process by identifying important imaging biomarkers that support the model’s predictions [41]. Saliency maps were employed by Baumgartner et al. (2019) to illustrate the brain MRI scan regions that had the greatest influence on CNN’s classification of Alzheimer’s disease. This method revealed the focus areas of the model, which matched known brain regions linked to disease [42]. Variations in scanner types and imaging techniques can impact the consistency of radiomic features. Up to 30% of frequently used radiomics features have poor repeatability across several CT scanners, according to Traverso et al. (2020) [31]. There is continuous work being done to create reliable features and standardize imaging procedures. It’s still hard to make sure ML models work properly for a variety of patient populations. For multi-institutional datasets, external validation is essential. This was shown by Park et al. (2020) by confirming the accuracy of their radiomics-based nasopharyngeal cancer recurrence prediction model in three separate cohorts [43]. While machine learning models yield encouraging results in research settings, there are obstacles in integrating them into clinical procedures. A path for implementing AI in radiology was presented by Langlotz et al. (2019), who emphasized the importance of workflow integration and prospective clinical trials [26].

### 7.3. Conclusions

The synergy between ML and radiomics promises to further progress personalized medicine as these issues are overcome and new methodologies emerge. This could result in more precise diagnoses, better treatment plans, and better patient outcomes across a range of medical disciplines.

## 8. Multimodal Integration and Enhanced Deep Learning Architectures in Radiomics

### 8.1. Introduction

The predictive modeling and analysis of medical images has been greatly improved by the combination of deep learning (DL) with radiomics. This section highlights current studies that show notable improvements in disease diagnosis, prognosis, and treatment response prediction. It also discusses state-of-the-art DL architectures, their specialized applications in radiomics, and the integration of multimodal data.

### 8.2. Discussion

CNNs are the mainstay of deep learning in radiomics because of their capacity to automatically extract intricate features from photos of medical conditions. The U-Net architecture was first presented by Ronneberger et al. (2015) and is now the industry standard for segmenting medical images [44]. With a Dice similarity value of 0.82, Zhou et al. (2019)’s adaptation of U-Net for pancreatic tumor segmentation in CT scans considerably outperformed conventional segmentation techniques [45]. ResNet was created by He et al. (2016) and introduces skip connections to enable considerably deeper networks [46]. A 3D ResNet was used by Ardila et al. (2019) for lung cancer screening, and the system performed as well in identifying malignant lung nodules as radiologists [47]. In medical imaging, GANs have demonstrated promise in resolving data scarcity problems. Wolterink et al. (2018) generated synthetic MRI scans from CT images using cycle-GANs for unpaired CT-to-MRI translation. This strategy might enhance training datasets and enhance the generalization of models [48]. Transformers were first created for natural language processing, but they have recently produced amazing results in medical imaging. UNETR, a transformer-based architecture for 3D medical picture segmentation, was presented by Hatamizadeh et al. in 2022. Their model demonstrated the potential of transformers in radiomics by achieving state-of-the-art performance on multi-organ segmentation tasks [49]. Predictive models that are more thorough and precise have been produced by the integration of radiomics with other data modalities. In oncology, combining genomic and radiomic data has produced encouraging outcomes. Xu et al. (2019) created a deep learning model to identify the status of EGFR mutations in lung adenocarcinomas by combining gene expression data with CT radiomics characteristics. Their model outperformed models based solely on genomes or radiomics, with an AUC of 0.85 [50]. Hosny et al. (2018) developed a deep learning model to predict patients’ 2-year overall survival by combining clinical factors and CT radiomics features. Compared to models that just used clinical data (0.57) or radiomics features (0.61), their model’s concordance index of 0.70 was much higher [38]. Nie et al. (2020) created a 3D CNN for brain tumor segmentation and survival prediction by fusing MRI and PET scans. Their multimodal strategy outperformed single-modality models in both tasks, proving the beneficial effects of mixing various imaging modalities [51]. Ensuring the interpretability of increasingly complicated DL models is crucial for their clinical application. Gradient-weighted Class Activation Mapping, or Grad-CAM, was first shown by Selvaraju et al. (2017) and has since gained widespread use in medical imaging [33]. To improve the transparency of the model, Gao et al. (2021) used Grad-CAM to depict the chest X-ray locations that had the greatest influence on their CNN’s COVID-19 diagnostic [52]. SHAP values were expanded to deep learning models by Lundberg and Lee (2017) [53]. In order to interpret a CNN trained on mammograms for breast cancer prediction, Binder et al. (2021) used Deep SHAP, which provided feature importance at both the pixel and semantic feature levels [54]. However, there are still a number of obstacles to overcome in order to fully integrate deep learning with radiomics. When compared to collections of natural images, medical imaging datasets are frequently smaller. To overcome this, research is being done on few-shot learning and transfer learning techniques. In their assessment of several transfer learning techniques for medical imaging, Cheplygina et al. (2019) emphasized how these techniques could enhance model performance even in the face of sparse data [55]. It is still difficult to guarantee that DL models function properly with different patient groups and imaging devices. By verifying their deep learning model for breast cancer risk prediction across several institutions and mammography suppliers, Yala et al. (2021) illustrated the significance of this [56]. Deep learning model training demands a large amount of processing power, particularly when using 3D medical pictures. To overcome this, approaches to distributed learning and cloud computing are being investigated. The viability of federated learning for brain tumor segmentation was shown by Sheller et al. (2020), enabling model training across institutions without requiring raw data sharing [12].

## 9. Regulatory and Clinical Adoption Considerations for Radiomics-Based CAD

### 9.1. Introduction

One possible method to improve clinical decision-making and medical image analysis is the synergistic combination of radiomics and CAD systems [2]. Those systems have predominantly depended on manually generated image characteristics. However, the integration of radiomics has broadened the scope of these systems’ functionality, permitting more thorough and individualized diagnostic evaluations [11]. By offering an abundance of quantitative imaging features that capture the subtle but clinically significant aspects of medical images, radiomics can improve CAD systems [12]. These features can reveal patterns and relationships in the data that might not be immediately visible to the human eye because they are obtained from different image processing and analysis approaches [57]. Researchers and physicians can create more reliable and accurate diagnostic models by including these radiomic variables into CAD algorithms. This could lead to improvements in the early diagnosis, characterisation, and monitoring of a variety of diseases [13].

### 9.2. Discussion

CAD systems based on radiomics have numerous potential advantages. Initially, by decreasing variability and enhancing diagnostic accuracy, these technologies can help radiologists and physicians make better educated and consistent decisions [14]. Furthermore, radiomics’ objective and quantitative character can offer information that complements more conventional qualitative evaluations, improving risk assessment, therapy scheduling, and illness management [6]. Additionally, radiomics-based CAD systems have the potential to enhance fairness and accessibility in healthcare. These technologies have the potential to lessen the burden on healthcare professionals by automating some diagnostic processes, especially in resource-constrained situations where there may not be as much access to skilled radiologists [58]. This may result in more accurate and timely diagnosis, which would eventually improve patient outcomes and guarantee more people have fair access to high-quality medical treatment [15]. But there are some obstacles and factors to take into account before radiomics-based CAD systems can be successfully used in clinical settings. Before approving a system for clinical usage, regulatory organizations, including the FDA and other governing bodies, have strict standards regarding the system’s effectiveness, safety, and validation [15,59]. In order to make sure that their systems fulfill the requirements for precision, resilience, and interpretability, researchers and developers must carefully negotiate these regulatory environments [60]. In addition, user acceptance, workflow integration, data privacy and security, and other aspects need to be carefully considered when integrating radiomics-based CAD systems into clinical workflow [12]. Patients must be informed about the advantages and drawbacks of these technologies, and clinicians must get training on how to use and interpret these systems appropriately [17].

### 9.3. Conclusions

Ongoing research and development initiatives are concentrated on resolving these issues and clearing the path for the broad clinical implementation of these revolutionary technologies as the fields of radiomics and CAD continue to advance. The medical imaging community can strive toward more individualized, precise, and equitable diagnostic solutions by utilizing radiomics and CAD, which will ultimately improve patient outcomes and advance precision medicine.

## 10. Future Directions and Limitations

Radiomics and AI coming together has great potential to advance personalized and precision medicine by increasing diagnostic accuracy and clinical decision-making [1,2]. The synergistic integration of these complimentary techniques is set to open up new avenues in the management of different diseases, ranging from early detection to personalized therapy planning, as they continue to evolve [11]. The creation of more reliable and accurate predictive models is one of the main ways that the integration of radiomics and AI might improve clinical decision-making and diagnostic accuracy. Clinicians can obtain deeper insights into the underlying pathophysiology of diseases by utilizing AI methods, such as machine learning and deep learning, to analyze and interpret the enormous array of quantitative imaging characteristics supplied by radiomics [57]. Better patient outcomes may follow from earlier and more accurate diagnosis, enhanced risk stratification, and more knowledgeable treatment choices [34]. Moreover, the improvement of precision and personalized medicine can be fueled by the combination of radiomics and AI [32,33]. AI-powered predictive models can create a more thorough image of each patient’s unique profile by merging radiomics data with other biomarkers, including genetic, clinical, and demographic data [5]. This can facilitate the development of a really personalized approach to healthcare by allowing the customization of diagnostic, prognostic, and therapeutic strategies to each patient’s particular traits [35]. For instance, the combination of radiomics and AI in oncology can be used to pinpoint imaging abnormalities linked to specific genetic or molecular markers. A more efficient and individualized approach to cancer care may be achieved by using this radiogenomic method to aid in the selection of targeted medicines and the tracking of treatment response [14]. The successful integration of AI and radiomics will depend on how the field develops and how problems with data standardization, model validation, and clinical deployment are resolved. In order to guarantee the security and effectiveness of these revolutionary technologies, ongoing efforts are concentrated on creating reliable and understandable AI models, encouraging interdisciplinary cooperation, and putting in place regulatory frameworks. However, AI models may also have some limitations in this context. One major issue is the need for large, high-quality datasets to train accurate models, which are often difficult to obtain in medical imaging. There’s also a risk of overfitting, where models perform well on training data but poorly on new, unseen data. Additionally, the complexity and lack of transparency in AI algorithms, especially deep learning models, can make it challenging for clinicians to interpret and trust the results. Data privacy and security concerns further complicate the integration of AI in clinical settings, as sensitive patient information must be protected. Lastly, regulatory hurdles and the need for standardized protocols can slow down the adoption of AI-driven radiomics in routine practice. In conclusion, clinical decision-making and medical imaging have undergone a revolutionary paradigm shift with the integration of AI and radiomics. Clinicians and researchers can open up new avenues for early disease detection, more precise risk stratification, and personalized treatment planning by utilizing the power of quantitative imaging and sophisticated computational techniques. This will ultimately pave the way for a future in which healthcare is more precise and individualized. The potential to enhance patient outcomes and revolutionize the provision of healthcare is enormous as these domains develop further.

## Figures and Tables

**Table 1:** Summary of key original studies reporting radiomic outcomes after AI integration in medical imaging. Abbreviations: CT: Computed Tomography; MRI: Magnetic Resonance Imaging; PET: Positron Emission Tomography; AI: Artificial Intelligence; ML: Machine Learning; DL: Deep Learning.

Study	Imaging Modality, Cancer Type	AI Technique	Radiomic Outcomes	Values	Advantages	Limitations
Aerts et al. (2014) [3]	CT Lung, Head and Neck	Radiomics, Machine Learning	Tumor phenotyping, Prognostic modeling	Quantitative imaging features associated with tumor genotype and clinical outcomes	Noninvasive, reproducible assessment of tumor characteristics	Requires large, well-annotated datasets for model training
Parmar et al. (2015) [11]	CT Lung	Radiomics, Machine Learning	Quantitative radiomic biomarkers	Identification of robust radiomic features predictive of clinical outcomes	Potential for risk stratification and personalized treatment planning	Feature selection and model validation remain challenging
Antropova et al. (2017) [12]	Mammography, Ultrasound, MRI Breast	Deep Feature Fusion	Breast cancer diagnosis	Improved breast cancer detection and characterization using multimodal imaging data	Leverages complementary information from different imaging modalities	Computational complexity and interpretability of deep learning models
Hosny et al. (2018) [13]	Multiple -	AI in Radiology	Potential of AI in radiology, challenges	Improved accuracy, efficiency, and consistency in medical image analysis	Opportunity to transform radiology practice and patient care	Concerns about data privacy, algorithmic bias, and ethical considerations
Wang et al. (2020) [14]	Multiple -	Radiomics, Deep Learning	Synergy of radiomics and deep learning	Leveraging the complementary strengths of radiomics and deep learning for clinical decision-making	Potential to enhance personalized medicine and precision diagnostics	Need for standardized protocols and interpretable hybrid models
Huang et al. (2016) [15]	CT Colorectal	Radiomics	Lymph node metastasis prediction in colorectal cancer	Preoperative prediction of lymph node involvement to guide treatment planning	Potential to improve surgical decision-making and avoid unnecessary procedures	Retrospective study design and need for prospective validation

## Data Availability

Not applicable.
